# Evidence of sex differences in cancer‐related cardiac complications in mouse models of pancreatic and liver cancer

**DOI:** 10.14814/phy2.15672

**Published:** 2023-04-26

**Authors:** Anna Gams, Alejandro Nevarez, Stephanie Perkail, Aileen Venegas, Sharon A. George, Tatiana Efimova, Igor R. Efimov

**Affiliations:** ^1^ Department of Biomedical Engineering The George Washington University Washington District of Columbia USA; ^2^ The George Washington Cancer Center The George Washington University School of Medicine and Health Sciences Washington District of Columbia USA; ^3^ Department of Biomedical Engineering Northwestern University Chicago Illinois USA; ^4^ Department of Anatomy and Cell Biology The George Washington University School of Medicine and Health Sciences Washington District of Columbia USA; ^5^ Department of Medicine Northwestern University Chicago Illinois USA

## Abstract

Abnormal heart rate variability (HRV) is commonly observed in cancer patients who have undergone targeted therapy and/or surgery, yet the effects of cancer itself on cardiac function remain underexplored. Specifically, there is limited knowledge about sex‐specific manifestations of HRV in cancer patients. Transgenic mouse models are widely used to study different types of cancer. Here, we aimed to investigate the sex‐specific effects of cancer on cardiac function using transgenic mouse models of pancreatic and liver cancers. This study used male and female transgenic mice with cancer and wild‐type controls. Cardiac function was assessed by recording electrocardiograms in conscious mice. RR intervals were detected to determine HRV using time and frequency domain analyses. Histological analysis with Masson's trichrome staining was performed to determine structural changes. In females, increased HRV was observed in both pancreatic and liver cancer‐bearing mice. In contrast, in males, increased HRV was observed only in the liver cancer group. Male pancreatic cancer mice demonstrated autonomic balance shift showing an increase in parasympathetic to sympathetic tone. The heart rate (HR) was higher in control and liver cancer male mice groups than in females. Histological analysis did not show significant sex differences but suggested a higher degree of remodeling in liver cancer mice than in control, specifically in the right atrium and left ventricle. This study revealed sex differences in cancer's HR modulation. Specifically, female cancer mice had lower median HR and higher HRV. These findings indicate that sex must be considered when using HRV as a cancer biomarker.

## Graphical abstract

Female mice developed higher heart rate variability (HRV) and lower heart rate in the liver and pancreatic cancer groups than control mice. The male pancreatic cancer group showed an autonomic balance shift toward increased parasympathetic tone. The male liver cancer group demonstrated increased HRV.

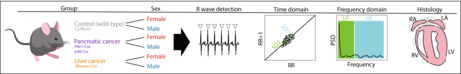

## 1 INTRODUCTION

Cardiovascular disease and cancer are the two leading causes of death worldwide, exceeding the COVID‐19 mortality rate. Both diseases show high rates of co‐occurrence and influence each other's progression. Specifically, over 60,000 people are diagnosed with pancreatic cancer each year. The pancreatic cancer rate in men is slightly higher than women. Pancreatic cancer is one of the most aggressive cancers ranking fourth in lethality, with a 5‐year survival rate of less than 10%, and has limited diagnostic and therapeutic options (Herreros‐Villanueva et al., 2012; Perkail et al., 2020). Several cases of heart failure have been reported in patients with pancreatic cancer (Byoun & Cho, 2020; Nunnery et al., 2020).

Worldwide, over 800,000 people are diagnosed with liver cancer each year. It is two‐to‐three times more prevalent in males than females. Similar to pancreatic cancer, cardiac complications, and cardiac tumor growth have been reported in patients with liver cancer (Marschall et al., 2021).

Over 19 clinical cancer studies, including pancreatic and liver cancers, showed decreased heart rate variability (HRV) correlated with cancer progression, suggesting possible autonomic dysfunction, potentially by a reduction in parasympathetic activity (Kloter et al., 2018). Increased HRV correlated with higher survival of cancer patients (Kloter et al., 2018). A current ongoing clinical trial is testing the use of changes in HRV as an early indicator of pancreatic cancer development (NCT04400903).

Our study used transgenic mouse models with several cancer‐specific mutations to induce pancreatic or liver cancer. These are well‐characterized genetically engineered mouse models in which tumors arise spontaneously and progress through well‐defined histopathological stages that closely recapitulate human disease, supporting the pathophysiological relevance of these murine models (Lee et al., 2016; Liu et al., 2022).

Two different pancreatic cancer mouse models driven by *Ptf1α* ^*Cre*^ and *PDX1* ^*Cre*^ were used in this study in the context of mutant *Kras* ^*G12D*^ and loss of tumor suppressor *Bap1* (*Ptf1α/Pdx1* ^*Cre*^ *; Bap1* ^*fl/fl*^ *; Kras* ^*G12D*^) (Perkail et al., 2020). The genetic model of pancreatic ductal adenocarcinoma (PDAC) involves conditional expression of proto‐oncogene *Kras* with activating point mutation (G12D) targeted specifically to the mouse pancreas due to Cre recombinase expression driven by pancreas‐specific promoters *Ptf1a* or *Pdx1* (Lee et al., 2016). As the progression to invasive and metastatic PDAC is known to be delayed in this model, with only a subset of mice developing cancer by 1 year of age, tumor suppressor *Bap1* was also deleted in the pancreas to accelerate cancer progression. As previously reported, *Ptf1α/Pdx1* ^*Cre*^ *; Bap1* ^*fl/fl*^ *; Kras* ^*G12D*^ mice, employed in the present study, develop locally invasive and metastatic PDAC at 10–15 weeks of age with high penetrance (Perkail et al., 2020). The liver cancer mouse model used in this study was driven by *Alb* ^*Cre*^ in the context of mutant *Kras* ^*G12D*^ (*Alb* ^*Cre*^ *; Kras* ^*G12D*^). The genetic model of primary liver cancer intrahepatic cholangiocarcinoma involves conditional expression of proto‐oncogene *Kras* with activating point mutation (G12D) targeted specifically to the mouse liver due to Cre recombinase expression driven by the liver‐specific Albumin‐Cre promoter. These mice develop multiple metastatic liver tumors by 36 weeks of age (O'Dell et al., 2012). BAP1 is histone H2A K119 de‐ubiquitinase and functions as a tumor suppressor in liver and pancreas. Thus, its knockout induces tumor growth (Baughman et al., 2016; Perkail et al., 2020). Mutation in *KRAS* (G12D) leads to activation of this potent oncogene (O'Hagan & Heyer, 2011). Permanent activation of oncogenic KRAS in the pancreas and liver has accelerated carcinogenesis by inducing cell proliferation, invasion, and survival (Buscail et al., 2020; Ikenoue et al., 2016; Perkail et al., 2020).

Recent evidence suggests cardiac remodeling and cancer progression are tightly linked (Avraham et al., 2020). This link goes both ways: heart failure or cardiovascular disease enhances existing (Koelwyn et al., 2020) or new (Avraham et al., 2020; Awwad et al., 2022) tumor growth, and the onset of cancer increases incidents of atrial fibrillation and other cardiac abnormalities (Conen et al., 2016; Guzzetti et al., 2002). In this study, we show the effects of cancer on the heart, specifically HRV and cardiac structure, using pancreatic and liver cancer models. We report sex‐specific cardiac responses in both cancer groups, with females having higher HRV and males demonstrating altered autonomic tone. These findings suggest that HRV can serve as a valuable cancer biomarker concerning cardiac function, but there is a significant sex dimorphism in the cancer‐heart disease axis.

## 2 METHODS

### 2.1 Mouse models

All animal protocols were approved by the Institutional Animal Care and Use Committee of The George Washington University and complied with the National Institutes of Health's Guide for the Care and Use of Laboratory Animals and the panel of Euthanasia of the American Veterinary Medical Association.

**FIGURE 1:**
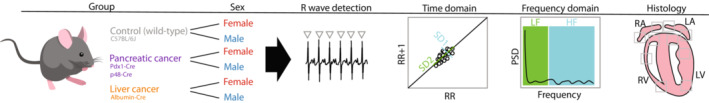
Schematic overview of the study workflow, electrocardiogram analysis, and histology.

Male and female transgenic mouse models with pancreatic cancer (male = 6; female = 10): *Ptf1α/Pdx1* ^*Cre*^ *; Bap1* ^*fl/fl*^ *; Kras* ^*G12D*^; and liver cancer (male = 6; female = 9): *Alb* ^*Cre*^ *; Kras* ^*G12D*^, were compared to control wild‐type mice (male = 5; female = 5): C57BL/6J (Figure 1). To note, *Ptf1α* ^*Cre*^ *; Bap1* ^*fl/fl*^ *; Kras* ^*G12D*^ (*n* = 5) and *Pdx1* ^*Cre*^ *; Bap1* ^*fl/fl*^ *; Kras* ^*G12D*^ (*n* = 11) were combined into one pancreatic cancer category since both regulate exocrine cell function and result in adenocarcinoma.

Electrocardiogram (ECG) was recorded from cancer mice before euthanasia between 4 and 46 weeks of age which is equivalent to 10–45 human years (Wang et al., 2020). Control mice ECGs were recorded every 2 weeks between 5 and 42 weeks of age. Each ECG recording was considered an independent data point. Control mice ECGs were age‐matched to cancer mice.

### 2.2 Masson's trichrome staining and histological data analysis

Mice were anesthetized with isoflurane vapors, and their hearts, pancreas, and liver were excised. Hearts were perfused with a cardioplegic solution and then with neutral‐buffered formalin. Hearts, pancreas, and liver were transferred to 70% ethanol after 24 h. Then, organs underwent embedding in paraffin, cross‐sectioning across chamber planes (long horizontal axis) and staining with Masson's trichrome stains.

The pancreas and liver were checked for tumor presence. Only mice that developed tumors were included in this study.

Stained heart samples were imaged at 20× magnification using Zeiss light microscope. The areas imaged included the left and right atrial free wall, appendage, left and right ventricular base, middle section, and apex.

A custom MATLAB code was used for color deconvolution to identify pink (myocytes), blue (collagen), and white (interstitial space) (Choi et al., 2021). The percentage of each color was quantified by calculating the number of pixels per color divided by a total number of pixels. The ratio of collagen to myocytes (blue/pink) was used to identify any structural changes.

### 2.3 ECG recording and analysis

ECGs were recorded from mice in the conscious state using non‐invasive emka Technologies ecgTUNNEL as described in Warhol et al. (2021). Mice were positioned in a restraining tunnel on four silver ECG electrodes. Mice were given 2–5 min to acclimate, and then ECGs were recorded for 5 min.

ECG traces were converted from proprietary emka files to .txt and analyzed with a custom MATLAB application. Due to mouse motion and breathing during the recording in the tunnel, the data were filtered for motion artifacts. First, ECG trace baseline drift was removed with Savitzky–Golay finite impulse response smoothing filter, then centered around zero and normalized. The R wave peaks were identified along with maximal overlap discrete wavelet transform (Park et al., 2009). The localization of both R wave peak and inverse wavelet peak was considered a true peak; the rest was discarded as a motion artifact.

Next, RR intervals were calculated along with median heart rate (HR). To obtain beat‐to‐beat HRV, both time‐domain and frequency‐domain analyses were implemented. For the time domain, consecutive RR intervals were used for Poincaré plot generation, RR_*n*_ versus RR_*n* + 1_. Dispersion of points perpendicular to the axis of the line‐of‐identity, SD1, represented short‐term variability and was calculated as $\mathrm{SD}1=\sqrt{\left(1/2\right){\text{SDSD}}^{2}}$, with SDSD being the standard deviation of the successive difference of RR intervals. Dispersion of points along the axis of the line‐of‐identity, SD2, representing long‐term variability, was calculated as $\mathrm{SD}2=\sqrt{2{\text{SDRR}}^{2}-\left(1/2\right){\text{SDSD}}^{2}}$, with SDRR being the standard deviation of RR interval (Karmakar et al., 2009; Shaffer & Ginsberg, 2017). The ratio of SD1/SD2 shows autonomic balance, with SD1 representing parasympathetic and SD2 sympathetic activity.

The frequency domain analysis was performed with Welch's power spectral density (PSD) estimate using RR intervals. Taking the integral of PSD versus frequency plot within low frequency (LF) and high frequency (HF) bands gave LF and HF power, respectively. The frequency range for LF, 0.1–1.5 Hz, represents sympathetic activity, whereas the frequency range for HF, 1.5–3 Hz, represents parasympathetic activity (Thireau et al., 2008). The ratio of LF/HF shows autonomic balance, sympathetic to parasympathetic. The ratio of SD1/SD2 in the time domain inversely correlates with the LF/HF in the frequency domain (Hsu et al., 2012).

### 2.4 Statistics

A Nonparametric Wilcoxon Rank Sum test with Bonferroni correction for multiple comparisons was used to determine statistical differences across groups at a significance level of *p* < 0.05. All data are shown as box‐and‐whiskers plots with upper and lower bars corresponding to the 25th and 75th percentiles and the middle bar to the median. An additional test to assess the interaction between cancer and sex was performed using Statistical analysis was performed using a two‐way analysis of variance (ANOVA) with Tukey's Honestly Significant Difference (Tukey's HSD) post hoc test for multiple comparison correction with significance at *p* < 0.05.

Post hoc power analysis was used to verify the statistical power of our study. The statistical test used was a two‐sample *t*‐test with an alpha level of 0.05. The effect size used for the power analysis was Cohen's *d* value, which was calculated to be >1.50 based on the observed means and standard deviations of the two groups. Our study had a power of over 0.8 which suggests that our sample size was sufficient to detect a moderate effect size with reasonable confidence.

## 3 RESULTS

### 3.1 Sex differences in time domain HRV in cancer mice

**FIGURE 2:**
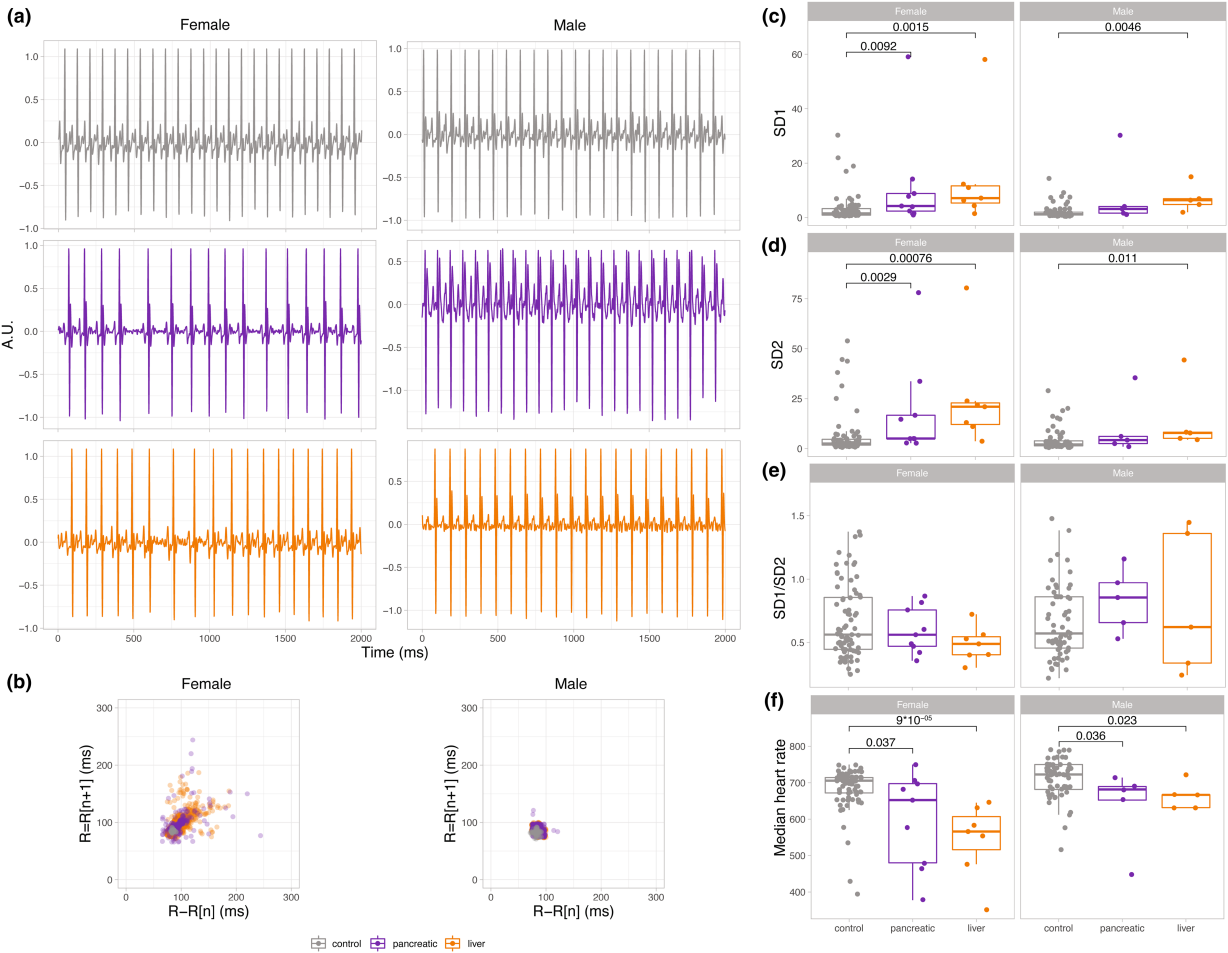
Time domain analysis of HRV reveals higher variability in female cancer mice. (a) Representative electrocardiogram traces of female mice (left panels) and male mice (right panels) showing control (gray), pancreatic cancer (purple), and liver cancer (orange) study groups. (b) Poincaré plots for female (left panel) and male (right panel) mice show HRV in control (gray), pancreatic cancer (purple), and liver cancer (orange) study groups. (c) SD1, (d) SD2, (e) SD1/SD2 ratios, and (f) median heart rate are shown for each group, with female mice on the left and male mice on the right panels. A Nonparametric Wilcoxon Rank Sum test was used to determine statistical differences by comparing pancreatic and liver cancer groups to control at a significance level of *p* < 0.05. All data are shown as box‐and‐whiskers plots with upper and lower bars corresponding to the 25th and 75th percentiles and the middle bar to the median. Sample sizes were the following: control: male = 5 (61 recordings), female = 5 (75 recordings); pancreatic cancer: male = 5 (5 recordings), female = 9 (9 recordings); liver cancer: male = 5 (5 recordings), female = 7 (7 recordings). A.U., arbitrary unit; HRV, heart rate variability; SD, standard deviation.

All cancer mice used in this study developed either pancreatic or liver tumors. Sex differences were assessed by comparing male and female mice of pancreatic and liver cancer groups to control. First, ECG traces showed large dispersion in beat‐to‐beat RR intervals in female cancer mice, suggesting increased HRV and smaller visual differences in male mice (Figure 2a). This was also confirmed by the Poincaré plot (Figure 2b). When comparing control and cancer mice of the same sex, SD1 and SD2 were significantly higher in both types of female cancer mice compared to control. Although the two female samples in the pancreatic and liver cancer groups appeared as outliers, further investigation found no reason to exclude them from the analysis. In males, SD1 and SD2 of liver cancer mice were significantly higher than in the control (Figure 2c,d). To summarize, these results demonstrate that liver cancer increased HRV in females and males, whereas pancreatic cancer increased HRV in females only.

Interestingly, no statistical differences were found in the autonomic balance of male and female control versus cancer mice (Figure 2e). Similarly, there were no differences in autonomic tone when comparing male to female mice (Figure S1b). Both sexes showed slower HR in cancer mice (Figure 2f), with significantly lower HR in females compared to males in control and liver cancer (Figure S1a). Moreover, there was a significant interaction between cancer and sex in the HR variable. Specifically, the two‐way ANOVA test showed a significant difference by group [*F* (2) = 15.8, *p* < 10^−6^] and by sex [*F* (1) = 10.04, *p* < 0.001], and the interaction between these factors was also significant. The Tukey post hoc test revealed significant pairwise differences between the control group and the pancreatic and liver cancer groups, as well as significant differences between male and female mice.

### 3.2 Sex differences in frequency domain HRV in cancer mice

**FIGURE 3:**
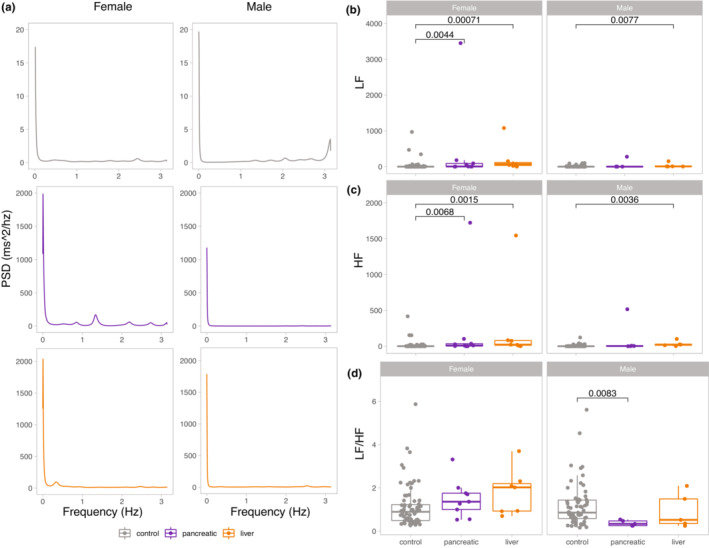
Frequency domain analysis of HRV reveals higher variability in female cancer mice. (a) Representative power spectral density (PSD) of female mice (left panels) and male mice (right panels) showing control (gray), pancreatic cancer (purple), and liver cancer (orange) study groups. (b) LF (0.1–1.5 Hz), (c) HF (1.5–4 Hz) bands, and (d) LF/HF ratios are shown for each group, with female mice on the left and male mice on the right panels. A Nonparametric Wilcoxon Rank Sum test was used to determine statistical differences by comparing pancreatic and liver cancer groups to control at a significance level of *p* < 0.05. All data are shown as box‐and‐whiskers plots with upper and lower bars corresponding to the 25th and 75th percentiles and the middle bar to the median. Sample sizes were the following: control: male = 5 (61 recordings), female = 5 (75 recordings); pancreatic cancer: male = 5 (5 recordings), female = 9 (9 recordings); liver cancer: male = 5 (5 recordings), female = 7 (7 recordings). HF, high frequency; LF, low frequency.

Frequency domain HRV analysis confirmed time domain findings. PSD traces showed higher variability (area under the curve) in female cancer mice compared with the control (Figure 3a). Female cancer mice had increased LF and HF power compared with the control (Figure 3b,c). Male mice only showed statistically higher LF and HF in liver cancer (Figure 3c). Additionally, the two‐way ANOVA test showed a statistically significant difference in LF by group [*F* (2) = 9.69, *p* < 10^−5^] and by sex [*F* (1) = 5.25, *p* = 0.023], and the interaction between these terms was also significant. A Tukey post hoc test revealed significant pairwise differences between control and cancer groups and significant differences between sexes. Similar results were found in HF statistical analysis as well.

Cancer did not alter LF/HF ratio in females while significantly reducing it in male mice with pancreatic cancer (Figure 3d). When comparing male to female mice, LF/HF ratio did not differ in control and liver cancer mice. In contrast, it was statistically higher in females than males in the pancreatic cancer group (Figure S1c). Taken together, liver cancer increased LF and HF power in females and males, while pancreatic cancer increased LF and HF only in females and decreased sympathetic to parasympathetic tone in male mice.

### 3.3 Structural remodeling

**FIGURE 4:**
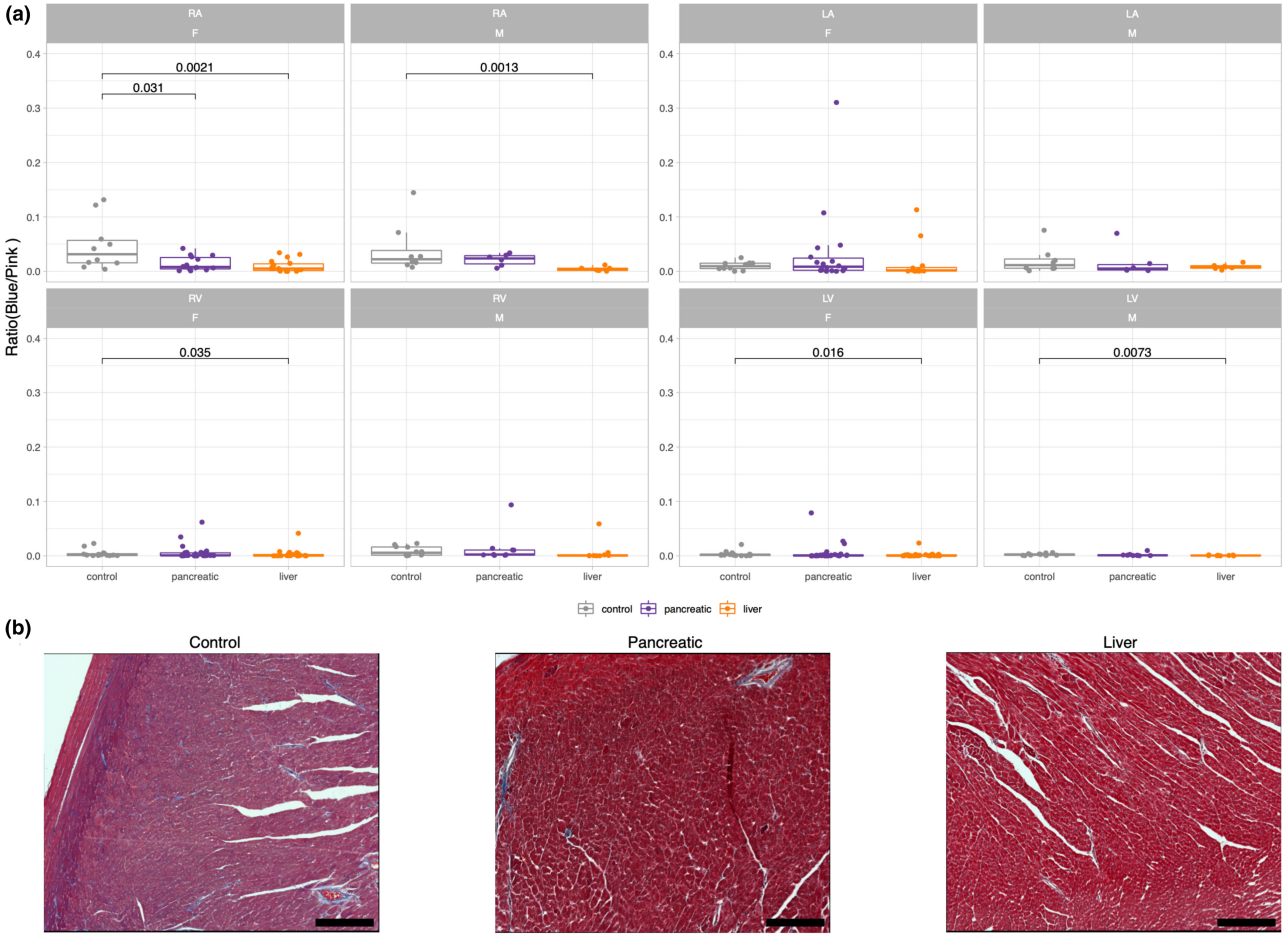
Structural changes quantification of Masson's trichrome staining. (a) The ratio of fibrosis (blue) to myocardium (pink) is shown for each chamber. A Nonparametric Wilcoxon Rank Sum test was used to determine statistical differences by comparing pancreatic and liver cancer groups to control at a significance level of *p* < 0.05. All data are shown as box‐and‐whiskers plots with upper and lower bars corresponding to the 25th and 75th percentiles and the middle bar to the median. Sample sizes were the following: control: male = 4 (RA—8, LA—8, RV—12, LV—12 images), female = 5 (RA—10, LA—10, RV—15, LV—15 images); pancreatic cancer: male = 3 (RA—6, LA—6, RV—9, LV—9 images), female = 10 (RA—1, LA—18, RV—30, LV—30 images); liver cancer: male = 3 (RA—6, LA—6, RV—9, LV—9 images), female = 9 (RA—18, LA—12, RV—27, LV—27 images). LA, left atrium; LV, left ventricle; RA, right atrium; RV, right ventricle. (b) Representative images of Masson's trichrome staining cross‐sections of LV. The scale bar size is 200 μm.

Masson's trichrome staining of the heart cross‐section that included every chamber was used to identify structural differences between control and cancer mice (Figure S2). To focus on collagen to myocyte proportions, ratios of those fractions were used. Interestingly, quantitative analysis revealed reduced blue/pink ratios in right atrium (RA) and left ventricle (LV) of liver cancer mice compared to control mice in both sexes (Figure 4). This reduction resulted from reduced fibrosis and not from a change in myocyte area (Figure S3). No significant differences in blue/pink ratios were observed in pancreatic mice versus control except for female RA. This analysis indicates cardiac remodeling in liver cancer compared to control, specifically in RA and LV, with no differences between sexes.

## 4 DISCUSSION

Numerous transgenic mouse models with different types of cancer have been developed over time. Usually, researchers study only the tumor paying little attention to cancer's effects on other organ functions. Recent evidence suggests that cancer and cardiovascular diseases are associated. Cardiac function modulation has been extensively studied in relation to cancer therapy but not the presence and progression of cancer itself (Narayan & Ky, 2018). We believe it is crucial to study cardiac function response to cancer development. ECG measurements are a cheap, non‐invasive, and effective way to study heart function. To the authors' knowledge, the present study is the first investigation of sex dimorphism in the cardiac function of mice with pancreatic and liver cancer.

Resting HR is a good indicator of the autonomic nervous system. A decrease in resting HR can be explained by higher parasympathetic activity compared to sympathetic or, in other words, increased vagal tone (Park et al., 2018). Both males and females showed a resting HR decrease in liver cancer groups suggesting vagal tone activation. Opposite to our findings, a higher resting HR predicts increased cancer mortality risk in humans (Gutierrez‐Martinez et al., 2021). Additionally, contrary to the fact that females have higher HR than males, both in humans and mice (Prabhavathi et al., 2014; Yang et al., 2007), our study showed the opposite phenomena across control and pancreatic liver groups. This is a curious discrepancy since several studies have reported in humans and conscious rats that healthy females have higher parasympathetic activity than males, which agrees with our findings (Korotkov, 2017; Yaghouby et al., 2020).

It has been shown that higher cancer survival has been associated with increased HRV (Hu et al., 2018; Kloter et al., 2018). Yet, high beat‐to‐beat variability is also associated with the development of arrhythmias (Kim et al., 2022). Thus, it is necessary to analyze ECG to understand the effect of tumor burden on the heart. HRV alone does not tell whether the cardiac function is normal; it should be linked with HR and autonomic tone (Sacha, 2014). For example, increased HRV and parasympathetic tone in bradycardic patients were cardioprotective (McLachlan et al., 2010).

Both time and frequency domain analyses showed increased HRV and decreased HR in cancer mice compared to control, particularly in females. Despite these significant changes in HRV and frequency response in female cancer mice, only male pancreatic cancer mice showed a statistically significant increase in parasympathetic activity compared to sympathetic in the frequency domain. These findings suggest that females were more affected by tumor burden exhibiting higher HRV with lower HR. Additionally, male mice with pancreatic cancer were less affected by tumor burden since they had higher HR, increased parasympathetic tone, and low HRV.

Cancer can cause various cardiovascular changes locally or systemically, increasing the risk of arrhythmias (Vyskočil et al., 2017). We also explored structural changes in cardiac tissue that support the modulation of cardiac function. Interestingly, a higher fibrosis percentage was detected in the control groups compared to liver cancer groups, potentially highlighting reduced fibrosis in these cancer models. The link between cancer and fibrosis can be established through transforming growth factor‐β (TGF‐β), a common activator of tumor growth and cardiac fibrosis (Parichatikanond et al., 2020). TGF‐β signaling inhibitors have been shown to have anticancer and antifibrotic properties. Yet, it requires further investigation into why fibrosis decreased with tumor growth in liver cancer‐bearing mice. Additionally, control and cancer groups showed no apparent sex differences in cardiac structure.

### 4.1 Clinical implications

HRV is a useful biomarker in assessing cancer progression and patients' outcomes (Kloter et al., 2018). Understanding trends of autonomic tone remodeling could allow for early detection, diagnosis, and sex‐specific predictions. Furthermore, this study shows that a combination of HRV analysis and HR provides a more complete picture of cancer's effects on cardiac function. Additionally, this study indicates that cardiac function used as a cancer biomarker needs to also consider the sex of a patient before interpreting the results. This study highlights that female heart function is more affected by pancreatic and liver cancer.

### 4.2 Limitations and future directions

This study provides the first evidence of sex dimorphism in the effects of cancer on cardiac function. Tracking cancer mice long‐term and continuously (via telemetry devices) would allow to closely study cancer progression and corresponding changes in cardiac function, such as arrhythmia incidence, HRV, and vagal tone modulation. Testing for the survival of animals with tumors could give an insight into stage 4 cancer cardiac function.

This study only determines the effects of liver and pancreatic cancers on heart function. Besides pancreatic (adenocarcinoma) and liver (intrahepatic cholangiocarcinoma) cancer, other cancer types should be studied; for example, the most common liver cancer hepatocellular carcinoma that results in very aggressive tumor growth. The effects of other cancer types will be a focus of future studies.

## 5 CONCLUSIONS

In this study, we investigated cardiac function in the presence of pancreatic and liver cancers. Monitoring mouse ECGs demonstrated a potential relationship between tumor burden and HRV, seen as larger dispersion on Poincaré plots and higher PSD traces, specifically in female mice. This study revealed that there are sex‐specific differences in cancer‐related HR modulation, with female mice showing lower median HR and higher HRV. These results suggest that HRV could be a valuable cancer biomarker, but sex differences must be considered.

## AUTHOR CONTRIBUTIONS

Anna Gams, Sharon A. George, Tatiana Efimova, and Igor R. Efimov conceptualized and designed the study, Stephanie Perkail provided animals, Anna Gams, Alejandro Nevarez, Stephanie Perkail, and Aileen Venegas conducted the experiments, Anna Gams and Alejandro Nevarez analyzed the data, Anna Gams and Alejandro Nevarez prepared the manuscript, and all authors edited and approved the manuscript.

## FUNDING INFORMATION

The study was funded by Fondation Leducq (Project: ‘RHYTHM’).

## CONFLICT OF INTEREST STATEMENT

The authors declare they have no conflicts of interest to report.

## Supporting information


Figure S1.

Figure S2.

Figure S3.
