# Supplementary figures and images for: Evidence of sex differences in cancer‐related cardiac complications in mouse models of pancreatic and liver cancer
Source: Physiol Rep. 2023 Apr 26;11(8):e15672. doi: 10.14814/phy2.15672 (PMC10133859; doi:10.14814/phy2.15672)

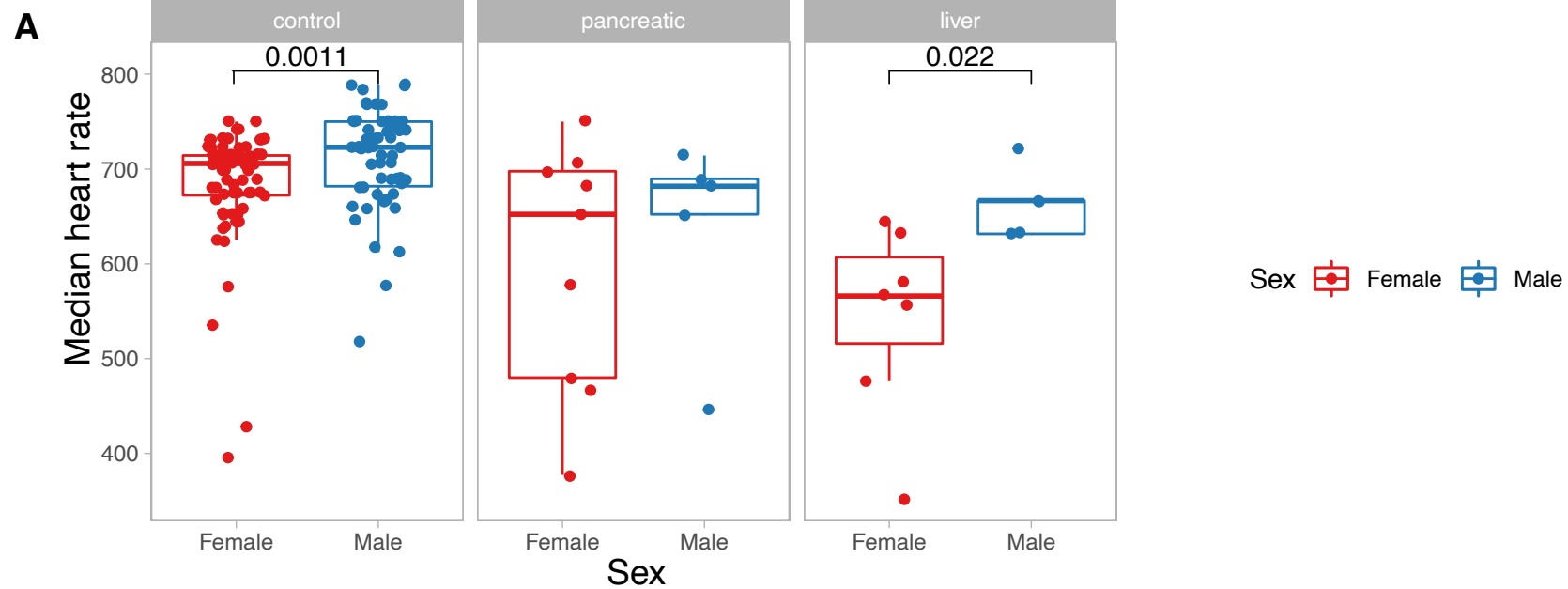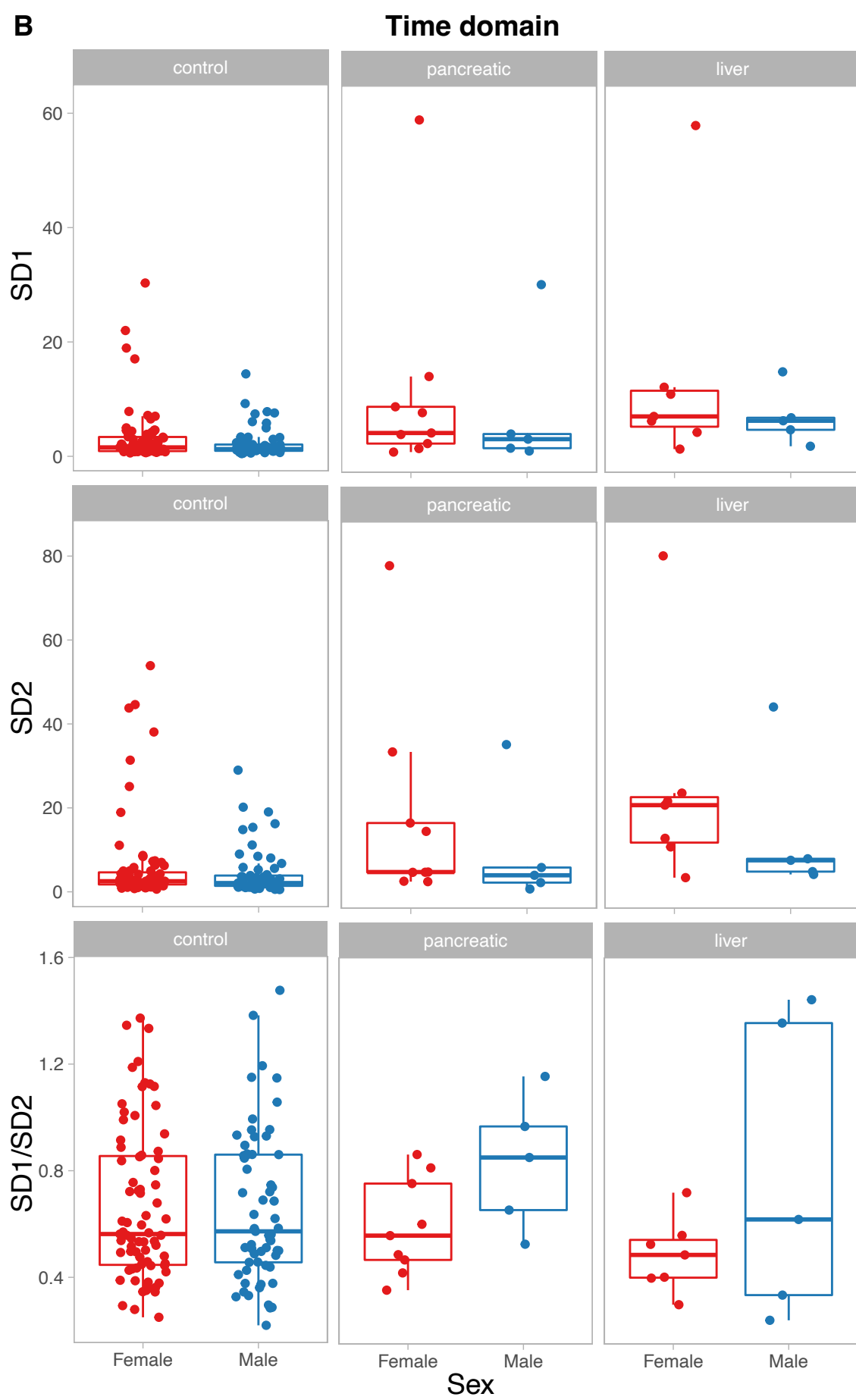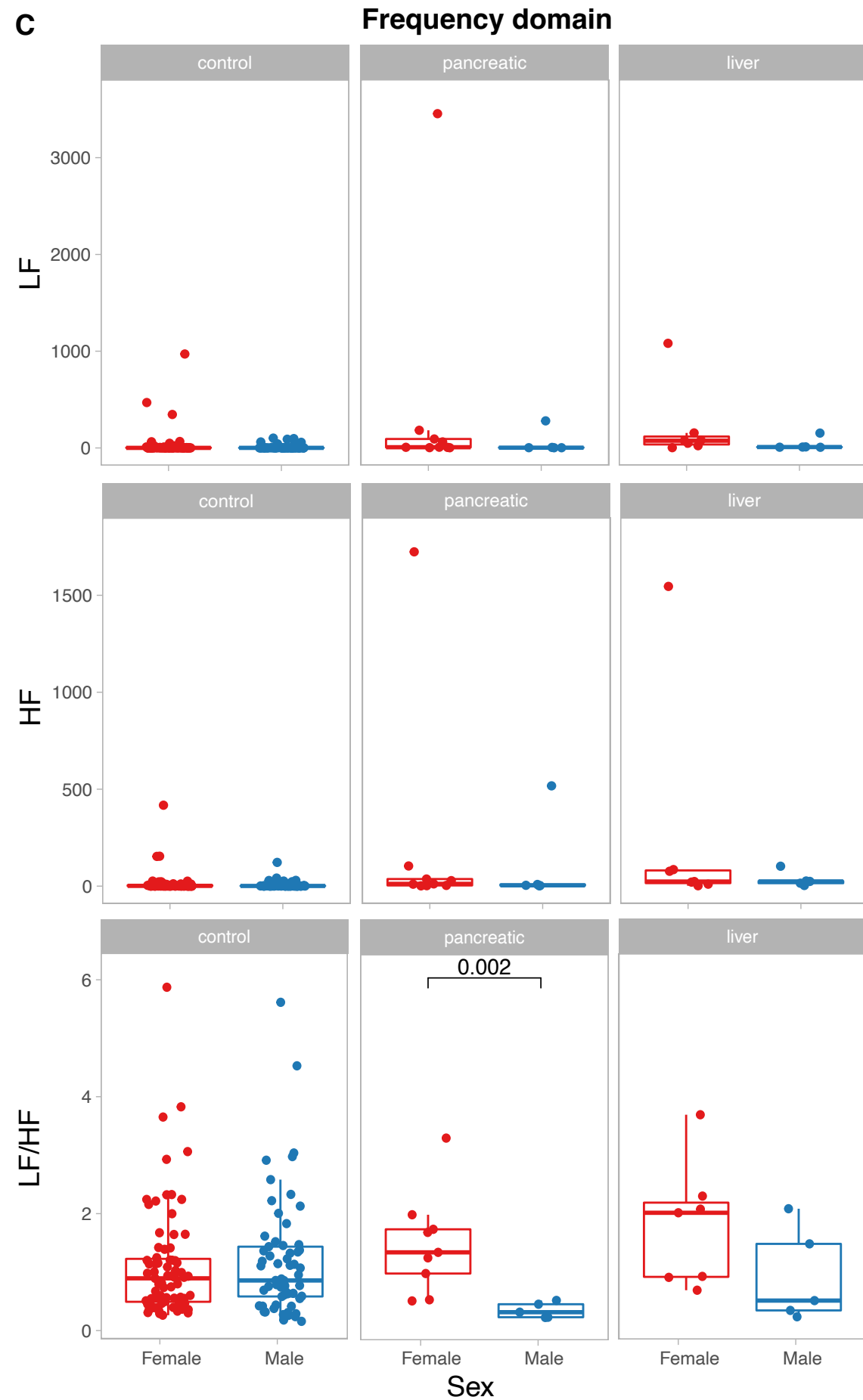

Supplement: Supplementary file 1 — Figure S1. [file PHY2-11-e15672-s003.pdf]

**A****Control**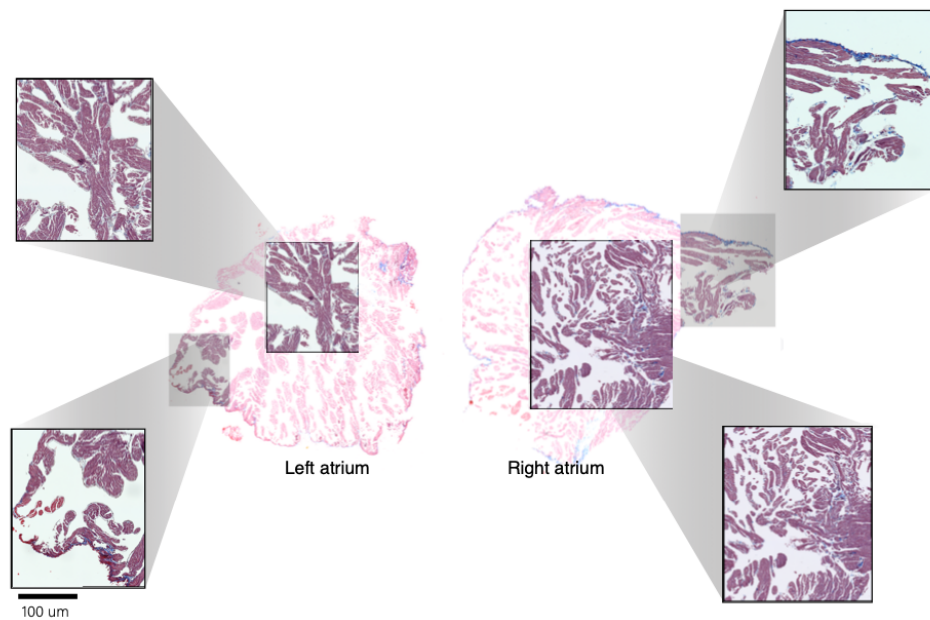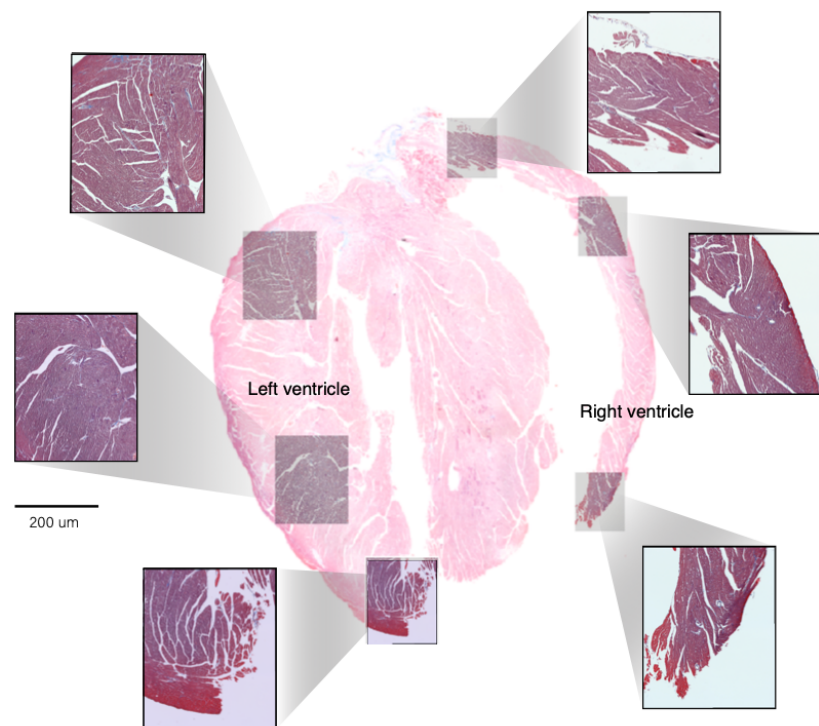**B****Pancreatic**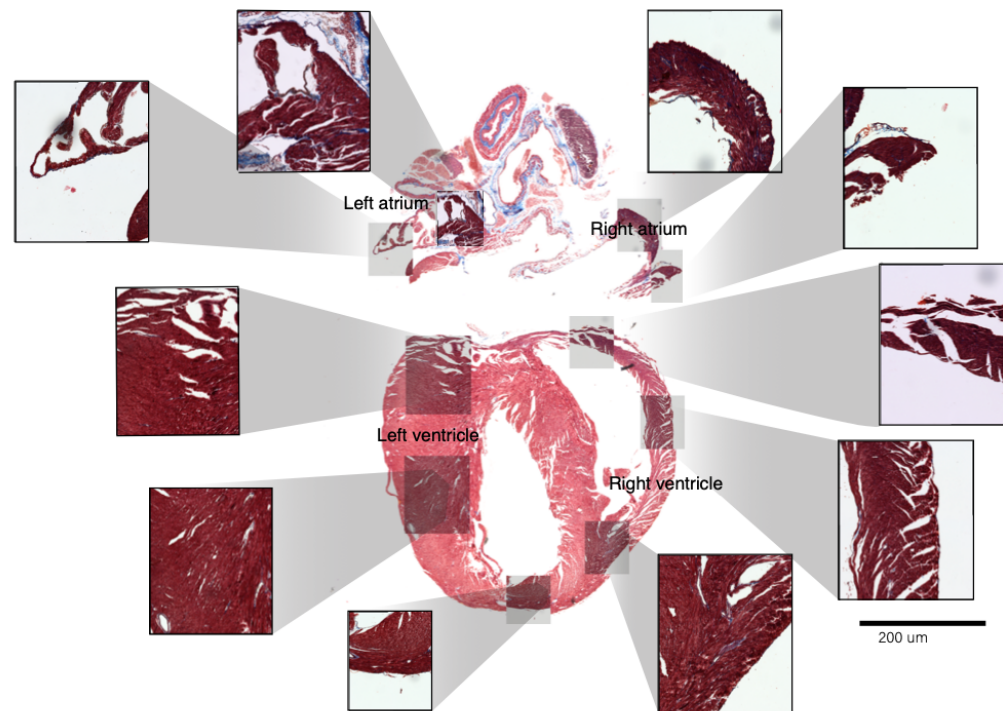**C****Liver**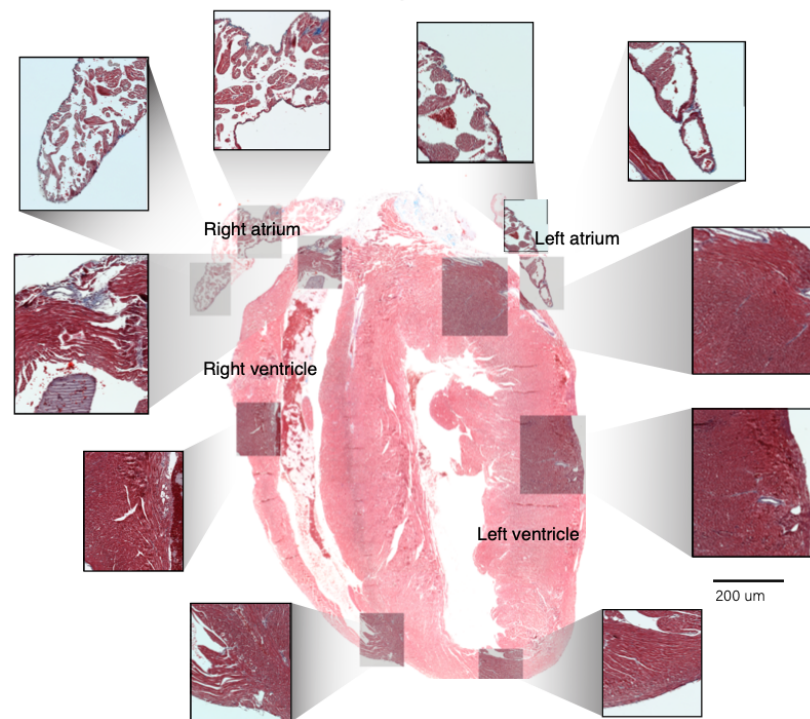

Supplement: Supplementary file 2 — Figure S2. [file PHY2-11-e15672-s001.pdf]

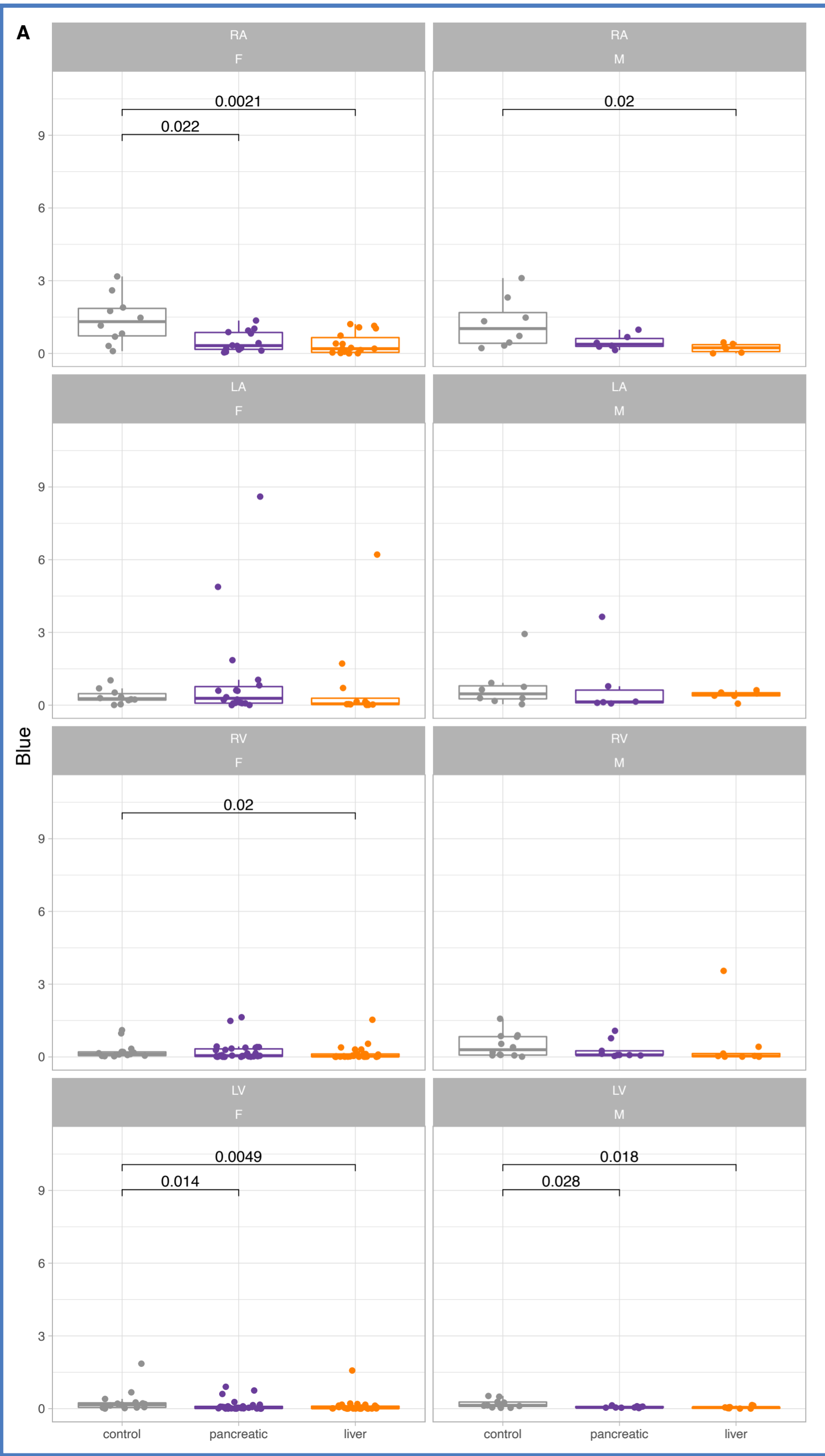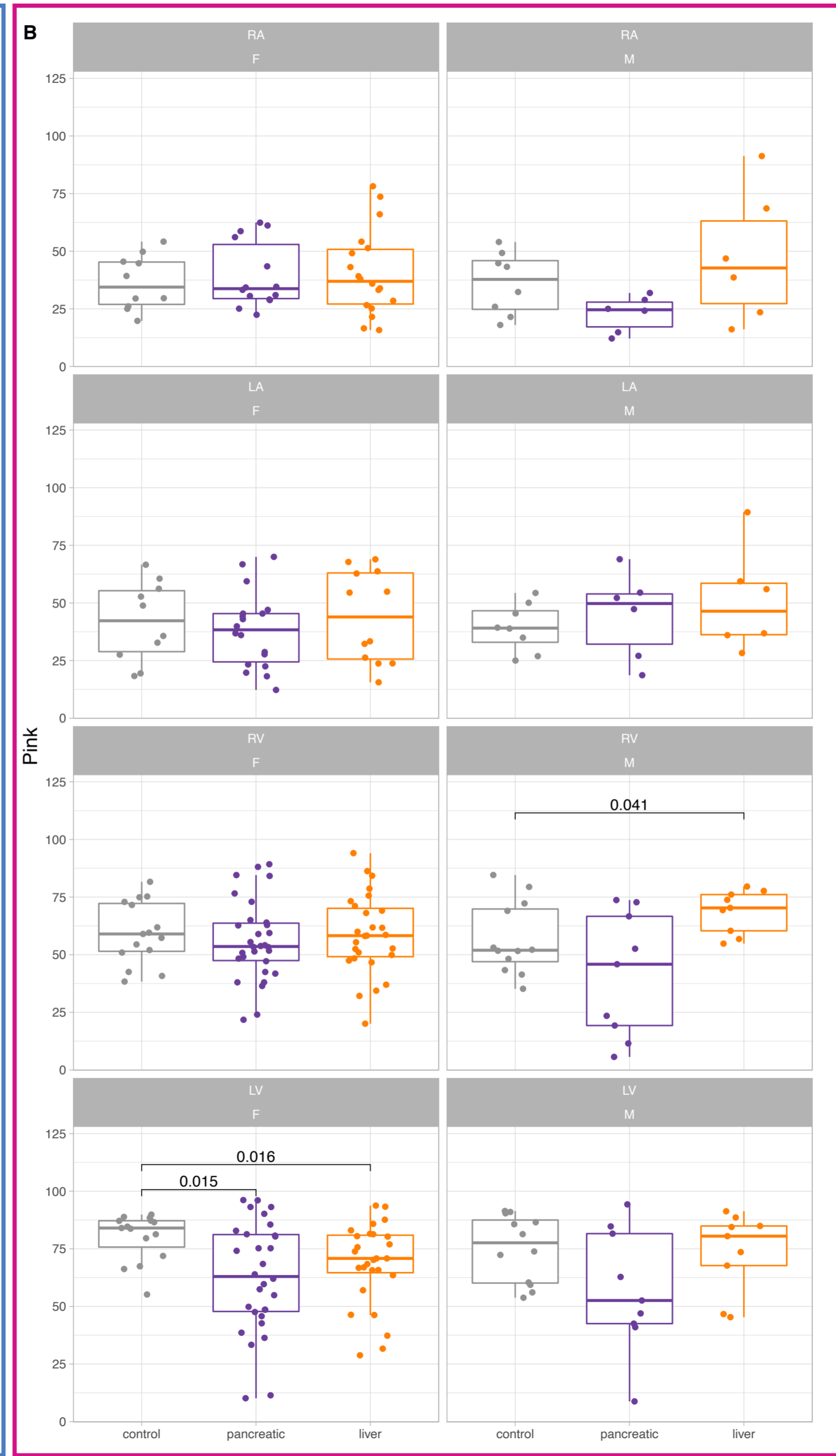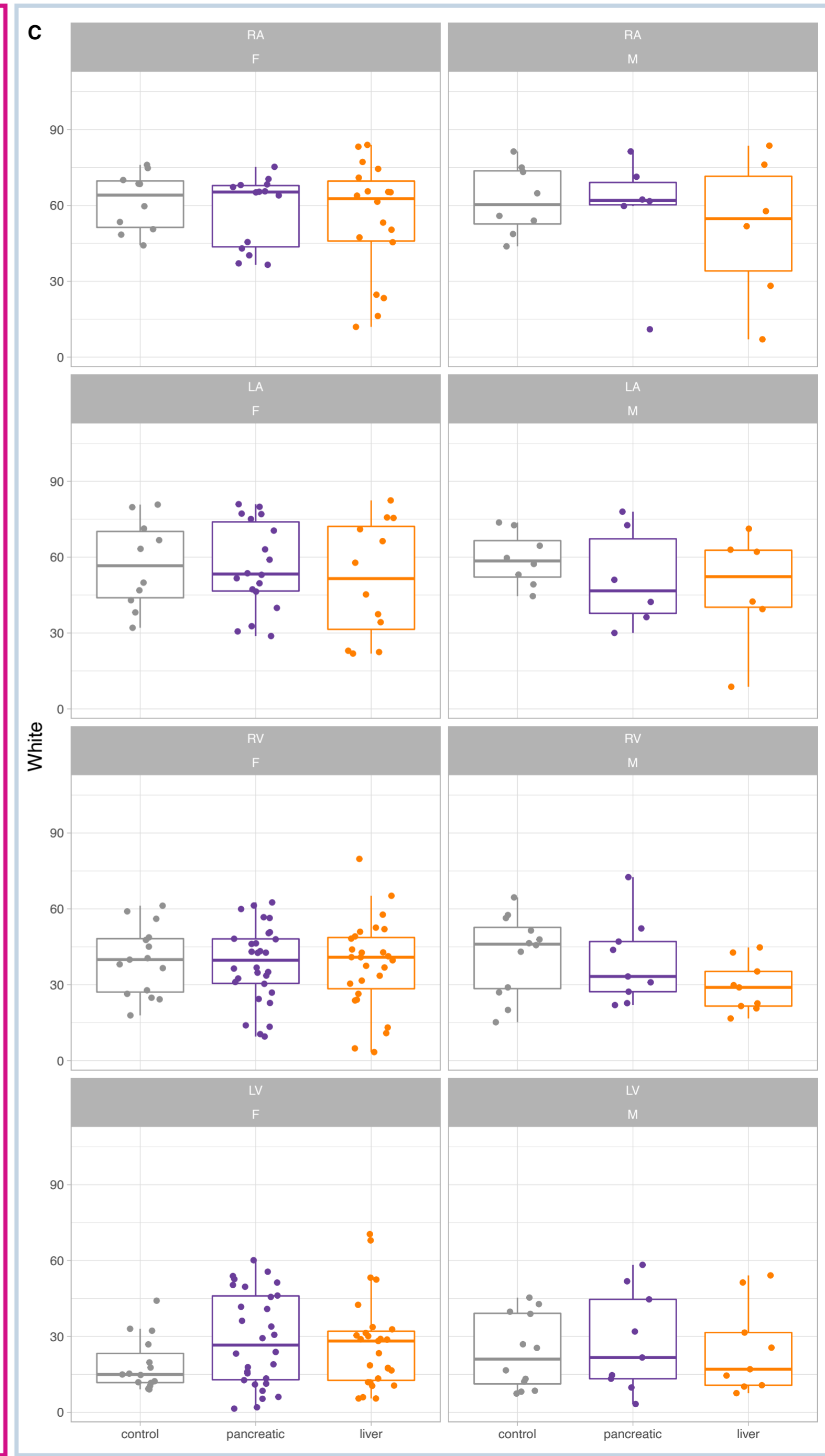

control pancreatic liver

Supplement: Supplementary file 3 — Figure S3. [file PHY2-11-e15672-s002.pdf]
